# Why do women choose to undergo oocyte aspiration without sedation or analgesia?

**DOI:** 10.1530/RAF-20-0064

**Published:** 2021-04-16

**Authors:** Daniella Gilboa, Liron Seidman, Polina Kimiagarov, Avia Noni, Ravid Doron, Daniel S Seidman

**Affiliations:** 1 IVF Unit, Assuta Medical Center, Tel Aviv, Tel-Aviv, Israel; 2 The Goldman Medical School at the Faculty of Health Sciences, Ben-Gurion University of the Negev, Beer-Sheva, Israel; 3 Department of Nursing, The Academic College of Tel-Aviv – Yaffo, Tel-Aviv, Israel; 4 Department of Education and Psychology, The Open University, Raanana, Israel; 5 Department of Obstetrics and Gynecology, Chaim Sheba Medical Center, affiliated to the Sackler Faculty of Medicine, Tel Aviv University, Tel Hashomer, Israel

**Keywords:** pain level, anxiety scores, fear of anesthesia, oocyte pick-up, sedation and analgesia

## Abstract

**Objective:**

Oocyte pick-up (OPU) is a painful but essential part of* in-vitro* fertilization (IVF) that is usually performed under sedation and analgesia (SaA). Our aim was to study that why some women decide to undergo OPU without SaA?

**Methods:**

This was a prospective study using patient questionnaires and the standardized 7-item generalized anxiety disorder (GAD-7) score. The patients were asked to assess the pain experienced during OPU using a visual analog scale (VAS). The study sample was a convenience sample of 100 healthy women undergoing OPU at our unit with or without SaA.

**Results:**

Women who chose to undergo OPU without SaA were significantly more likely to express the fear of anesthesia. A high pain score (VAS ≥ 6) was reported by significantly more patients who underwent OPU without SaA than with SaA. Yet, 98% of the patients who underwent OPU without SaA stated that in future IVF cycles, they would still choose to undergo OPU without SaA. More patients had high anxiety scores among those who underwent OPU with than without SaA.

**Conclusions:**

Women who chose to undergo OPU without SaA reported more often fear of anesthesia. Although these women experienced significantly more pain during OPU, almost all of them suggested that they would still choose to undergo OPU without SaA. Increased anxiety, as expressed by higher GAD-7 scores, was not associated with a tendency to choose SaA during OPU. The option of OPU without SaA seems to be an acceptable option for selected women.

**Lay summary:**

Egg retrieval from the ovaries is a painful part of* in vitro* fertilization (IVF). It is, therefore, usually performed under sedation and pain relief (analgesia). The aim of this study was to investigate: Why some women decide to undergo egg retrieval without sedation? We prospectively studied 100 women using patient questionnaires and standardized scores in order to measure patient's pain and anxiety levels. We found that women who chose to undergo egg retrieval without sedation were significantly more likely to express fear of anesthesia. As expected, women who decided to forgo sedation experienced more pain during egg retrieval, yet, 98% of them decided that in future IVF cycles, they would still choose to undergo egg retrieval without sedation. Surprisingly, women who had high anxiety scores were not more likely to ask for sedation during egg retrieval. The option to undergo egg retrieval without sedation during IVF seems to be acceptable for some women.

## Introduction

The question of whether to perform ovum pick-up (OPU), under sedation and analgesia (SaA), is of practical importance since some patients fear the anesthesia more than they fear the procedure itself. For such patients, a reassurance that the procedure can be performed without anesthesia is of great significance. Moreover, for some high-risk patients, with contraindications for anesthesia, the option of undergoing OPU without anesthesia may be key for allowing them to undergo IVF and become biological mothers. Performing OPU without anesthesia may also have cost- and time-saving implications for the IVF clinic.

When women undergo OPU they are often anxious and usually experience mild to moderate pain caused by the puncture of the vaginal wall and ovarian capsule with the OPU needle (Sharma *et al.* 2015, Kwan *et al.* 2018, Iduna Antigoni Buisman *et al.* 2020). Since OPU is often repeated several times to collect the maximal number of oocytes, it is usually performed under SaA (Sharma *et al.* 2015, Kwan *et al.* 2018).

A recent Cochrane systemic review examined the literature, studying the various methods of conscious SaA used for pain relief during OPU in IVF procedures (Kwan *et al.* 2018). Evidence collected from 21 randomized controlled trials did not support one particular method or technique of SaA over another in providing effective pain relief during and after OPU. Since all the numerous SaA approaches and techniques reviewed seemed to be acceptable and were associated with a high degree of satisfaction. The authors concluded that the optimal method should be individualized depending on the preferences of both the women and the clinicians and available resources (Kwan *et al.* 2018).

In recent years, we have encountered a growing number of IVF patients who choose to avoid SaA during OPU. Various reasons can explain the decision to give up the option of undergoing OPU under SaA. Apparently, some women are concerned with the drugs used during SaA, and are looking for a more natural course of IVF treatment (Matsota *et al.* 2015, Urfalioğlu & Yaylali 2016, Rolland *et al.* 2017). Since all patients undergoing OPU at our IVF center are routinely offered SaA by an experienced anesthetist free of charge, it seems clear that financial considerations do not contribute significantly to our patients’ decision to give up SaA.

The reasons leading women to undergo OPU without SaA have not been studied extensively (Greenberger *et al.* 2020). Moreover, little has been published to date regarding the related pain and overall acceptance of OPU without anesthesia.

The aim of the present study was to assess why some women decide to abstain from SaA during OPU and whether anxiety levels are associated with the patients’ decision to undergo OPU with or without SaA.

## Materials and methods

We prospectively obtained sociodemographic and outcome data using validated questionnaires, modified for IVF patients. Prior to completing the questionnaires, all patients received an explanation of the study’s goals and signed an informed consent form. The study was approved by the Assuta Medical Center, Tel Aviv, human investigation review board (IRB).

### Study design

This was a prospective study of 100 healthy women who underwent IVF at our unit during September through December 2016. Patients who agreed to participate in our study were asked to complete the study questionnaires before and after OPU. The patients were recruited in a 1:1 ratio according to their decision to undergo OPU with or without SaA. Data regarding the number of oocytes obtained per OPU and the pregnancy rates following the IVF treatment were collected.

### Questionnaire design and validation

The patients completed the questionnaires during two separate meetings. The first encounter took place at least 1 h before the IVF procedure. All patients received a full explanation of the study and signed an informed consent. They were then asked to complete a Hebrew version of the standardized generalized anxiety disorder (GAD-7) validated 7-item self-report questionnaire. The GAD-7 is a reliable and validated metrics (Spitzer *et al.* 2006) in assessing symptoms of anxiety. We, therefore, employed this metrics in our assessment of anxiety symptoms in IVF patients over the 2 weeks preceding the OPU (Spitzer *et al.* 2006). We used this measure to classify participants in the following categories, per GAD-7 cut-off scores (total scores range from 0 to 21): minimal anxiety (0–5), moderate anxiety (10–14) and severe anxiety (10 and over).

During the second encounter, once the patient was fully alert, and at least 1 h following the OPU, the patients were asked to assess the pain experienced during the OPU using a visual analog scale (VAS), graded 0–10. Non-narcotic analgesic pills were offered upon request after completion of the VAS scores. The participants were also asked after the OPU whether they regretted undergoing the procedure with or without anesthesia, and whether they would recommend to a friend to undergo OPU with or without anesthesia. Additional outcome data were obtained from the patients’ charts.

### OPU technique

Oocyte aspiration was performed at our dedicated IVF operating room at Assuta Medical Center, an academically affiliated private non-profit medical center, on an outpatient basis. Patients were not given premedication. The OPU procedures were performed by an experienced physician (DSS) who was not aware of the collected data. A reduced (17-gauge body, 20-gauge tip) OPU needle (Swemed Sense,Vitrolife Sweden AB, Gothenburg) was used in all procedures.

### Anesthesia

Premedication was not administered to any of the patients before undergoing OPU. Patients electing to undergo OPU without SaA were fully conscious and received no pain medication and no local anesthesia. Patients who chose to undergo deep SaA received total –i.v. anesthesia with propofol and fentanyl according to their body weight. They were spontaneously breathing throughout the OPU but were not aware of the procedure.

An anesthesiologist was available, free of charge, as needed, during all OPUs, regardless of the patients’ decision on the use of SaA.

### Power analysis

We assumed that to detect an effect size of 0.6 on the GAD-7 score, a sample size of 50 in each group will have an 80% power, using a 2-group *t*-test with a 0.05 two-sided significance level.

### Statistical analysis

Descriptive statistics and 95% CI were summarized and are presented by treatment group. Statistical significance was determined using *t*- test or Fisher’s exact test depending on the distribution of the outcome measures. All data summaries and statistical analyses were generated using SAS^®^ version 9.3.

## Results

The IVF patients who elected to undergo OPU with or without SaA did not differ significantly in their demographic characteristics, including mean age, marital status, level of education, self-stated financial status and area of residence (Table 1). All were Jewish and Caucasian ethnicity. The median number of oocytes aspirated was similar among the patient who underwent OPU with or without SaA, four (range 1–26)– vs five (range 1–41) (Table 2). However, patients who underwent OPU without SaA were significantly more likely to have no more than two oocytes aspirated, 46% vs 8%, *P* < 0.05. The pregnancy rates were not statistically different in both groups (Table 2).

**Table 1:** Characteristics of IVF patients undergoing OPU with or without sedation and analgesia. Data are presented as *n* (%).

	**SaA during OPU** (*n* = 50)	**No SaA during OPU** (*n* = 50)
Age (years), mean ± s.d.	38.7 ± 4.2	40.8 ± 3.4
Marital status		
Divorced	4 (8.2%)	4 (8%)
Married	28 (57.1%)	25 (50%)
Single	17 (34.7%)	21 (42%)
Education level		
High school	13 (26%)	5 (10%)
University	37 (74%)	45 (90%)
Financial status		
Average income	24 (48%)	32 (64%)
Above average income	26 (52%)	18 (36%)
Place of residence		
Center	35 (70%)	35 (70%)
North	3 (6%)	1 (2%)
Sharon/South	12 (24%)	14 (28%)

OPU, oocyte pick-up; SaA, sedation and analgesia.

**Table 2:** Outcome of IVF in patients who underwent OPU with or without sedation and analgesia.

	**SaA during OPU** (*n* = 50)	**No SaA during OPU** (*n* = 50)	*P-***value**
Number of previous OPU cycle			
0–3	22 (44%)	15 (30%)	ns
4–7	21 (42%)	17 (34%)	ns
8–10	4 (8%)	9 (18%)	ns
>10	3 (6%)	9 (18%)	ns
Number of oocytes aspirated			
0	1 (2%)	3 (6%)	ns
1	2 (4%)	13 (26%)	<0.05
2	2 (4%)	10 (20%)	<0.05
≥3	45 (90%)	24 (48%)	<0.05
Biochemical pregnancy	21 (42%)	17 (34%)	ns

Biochemical pregnancy: a pregnancy diagnosed only by the detection of beta hCG in serum or urine (Zegers-Hochschild *et al.*, 2017).

OPU, oocyte pick-up; SaA, sedation and analgesia.

More patients had high anxiety scores among those who underwent OPU with SaA than without SaA, 12% vs 6%, respectively, a statistically non-significant difference (Table 3). Women who chose to undergo OPU without SaA were significantly more likely to express fear of anesthesia, 58% vs 38% for patients who underwent SaA, *P* < 0.05.

**Table 3:** Anxiety and pain scores in IVF patients who underwent OPU with or without sedation and analgesia.

	**SaA during OPU** (*n* = 50)	**No SaA during OPU** (*n* = 50)	***P*-value**
Ever used antianxiety meds	13 (26%)	4 (8%)	<0.05
Fear of anesthesia	19 (38%)	29 (58%)	<0.05
Pain scale (VAS) during OPU			
Low (VAS < 6)	47 (94%)	38 (76%)	<0.05
High (VAS ≥ 6)	3 (6%)	12 (24%)	

OPU, oocyte pick-up; SaA, sedation and analgesia; VAS, visual analog scale.

None of the patients was asked to stop the OPU procedure prematurely due to pain or asked for SaA after declining it initially. As expected, the pain score during OPU was more frequently high (VAS ≥ 6) in patients who underwent OPU without SaA than with SaA, 24% vs 6%, a statistically significant difference, *P* < 0.05. There was no difference in the proportion of IVF patients that were afraid of pain, expressed high anger levels or reported taking antianxiety meds on the day of OPU (Table 3).

The attitudes toward anesthesia in IVF patients who underwent OPU with or without SaA are shown in Table 4.

**Table 4:** Attitudes toward anesthesia before and after anesthesia in IVF patients who underwent OPU with or without sedation and analgesia.

	**SaA during OPU** (*n* = 50)	**No SaA during OPU** (*n* = 50)	***P-*value**
Previously undergone SaA	39 (78%)	37 (74%)	Ns
Knew about the option of OPU without SaA	42 (84%)	47 (94%)	Ns
OPU without SaA advised	3 (6%)	29 (58%)	<0.05
Would like to be awake during OPU	11 (22%)	25 (50%)	<0.05
No regret undergoing OPU with SaA	46 (92%)		
No regret undergoing OPU without SaA		49 (98%)	
Would recommend SaA during OPU	44 (88%)	12 (24%)	<0.05

## Discussion

OPU is usually performed under SaA (Sharma *et al.* 2015, Kwan *et al.* 2018). Some IVF patients, however, elect to undergo the painful procedure without any form of anesthesia or sedation (Greenberger *et al.* 2020). In our study, only one patient (2%) in the group that chose to undergo OPU without SaA, regrated her choice. This shows the high acceptability of OPU without SaA among women who selected this option.

Our findings show that the major determinants supporting the decision to undergo the procedure without SaA were the number of oocytes aspirated and fear of the general anesthesia. Of the patients who chose to undergo OPU without SaA, 52% had less than three oocytes aspirated vs 10% of those who underwent OPU with SaA. Since the number of oocytes retrieved is strongly related to the duration of the procedure and thus to the expected degree of pain (Ng *et al.* 2001, Garg & Dali 2007), it is reasonable to assume that this is not a chance finding. Most of the patients who chose to undergo OPU without SaA reported that they were advised regarding this option (Table 4). We believe that many of these patients were advised, based on the small number of follicles observed prior to OPU, that they are likely to undergo a shorter procedure and thus experience less pain.

It was previously suggested that some patients may prefer to undergo OPU without SaA due to concerns regarding possible detrimental effects of sedation and analgesia medications on reproductive outcomes (Garg & Dali 2007, Matsota *et al.* 2015, Kwan *et al.* 2018). Many anesthetic drugs were detected in the follicular fluid and may possibly interfere with oocyte fertilization and implantation (Garg & Dali 2007, Sharma *et al.* 2015). Although there is apparently no well-based evidence for serious toxicity in humans (Garg & Dali 2007), currently some IVF patients prefer a more ‘natural’ approach, thus minimizing the use of all nonessential drugs. Electing to undergo OPU without SaA seems to be part of this minimalistic approach.

IVF treatment is known to be associated with increased levels of anxiety (Schaller *et al.* 2016). The stress and anxiety are related to possible failure to achieve a successful pregnancy despite the investment of many resources during the process (Schaller *et al.* 2016). However, some of the anxiety is directly concerned with the health risks the women must take such as ‘bleeding or infection after the oocyte aspiration’ (Schaller *et al.* 2016). In our study, patient's anxiety levels were measured by the GAD questionnaire. Fear of pain and anger levels in the GOD questionnaire apparently had little influence of the decision on SaA. Stening *et al.* (2012) showed that pain perception and tolerance in women undergoing IVF did not vary, despite the dramatic changes in 17β-estradiol levels induced by the treatment regimen. They suggested that changes in estrogen levels have little influence on pain sensitivity in humans, contrary to experimental animals.

Concern regarding pain during OPU is usually overcome by the widespread use of SaA. However, our study supports the notion that for some patients the use of SaA creates a new form of concern. Our results show that fear of anesthesia was more strongly related to the decision to undergo OPU without SaA then patients’ anxiety levels. However, among patients who declined SaA prior to OPU, none asked to stop the OPU procedure prematurely due to pain or changed their mind during the procedure.

OPU is associated with a significant degree of pain. However, three quarters of our patients reported experiencing only mild to moderate pain when undergoing OPU without SaA. This could be attributed at least in part to the use of improved newly designed OPU needles with a thin tip (Wikland *et al.* 2011, Nakagawa *et al.* 2015, Iduna Antigoni Buisman *et al.* 2020). Oocyte aspiration performed with the newly designed thinner-tipped needles, like the 19-gauge needle used in the present study, were shown to be associated with significantly less overall pain (Wikland *et al.* 2011, Nakagawa *et al.* 2015, Iduna Antigoni Buisman *et al.* 2020).

In our study, women made their decision regarding whether to undergo OPU with or without SaA prior to recruitment. This means that our study reflects the objective decision of the women before enrollment. The inherent selection bias is acknowledged, however, it does not interfere with the goal of this study which was to extend our understanding of why some women decide to undergo OPU without SaA. Since all patients undergoing OPU at our center are routinely offered SaA at no extra cost, financial concerns could not have been a major consideration for our study participants.

We conclude that women who chose to undergo OPU without SaA reported more often fear of anesthesia and were more likely to have two or fewer oocytes aspirated. Although women who underwent OPU without SaA experienced significantly more pain during OPU, almost all of them suggested that they would choose to undergo OPU again without SaA. Increased anxiety, as expressed by higher GAD-7 scores, was not associated with a significant tendency to choose SaA during OPU. The option of OPU without SaA seems to be an agreeable option for selected women. Moreover, our data suggest that women who are considered unsuitable for SaA can be reassured that most women find this option acceptable and associated with a reasonable level of pain.

## Declaration of interest

The authors declare that there is no conflict of interest that could be perceived as prejudicing the impartiality of the research reported.

## Author contribution statement

D G and D S S conceived the research question. D G, D S S and D R verified the analytical methods. P K and A N recruited the patients, administered the study questionnaires and asked the patients to assess the pain experienced. D G performed the statistical analysis. D G, L S, D R and D S S supervised the findings of this work and contributed to the interpretation of the results. D G wrote the manuscript with support from D R, L S and DS.S. All authors discussed the results and contributed to the final manuscript.

## Presentation

This work was presented as a poster at the ASRM 2017 Scientific Meeting, San Antonio, Texas, October 29, 2017.
